# Endogenous estrogens—breast cancer and chemoprevention

**DOI:** 10.1007/s43440-021-00317-0

**Published:** 2021-08-30

**Authors:** Beata Starek-Świechowicz, Bogusława Budziszewska, Andrzej Starek

**Affiliations:** 1 Department of Biochemical Toxicology, Chair of Toxicology, Medical College, Jagiellonian University, Medyczna 9, 30-688 Kraków, Poland; 2 Department of Experimental Neuroendocrinology, Institute of Pharmacology, Polish Academy of Sciences, Smętna 12, 31-343 Kraków, Poland

**Keywords:** Breast cancer, Endogenous estrogens, Estrogen metabolism, Chemopreventive agents

## Abstract

Breast cancer is the most common female malignancy and the second leading cause of cancer related deaths. It is estimated that about 40% of all cancer in women is hormonally mediated. Both estrogens and androgens play critical roles in the initiation and development of breast cancer. Estrogens influence normal physiological growth, proliferation, and differentiation of breast tissues, as well as the development and progression of breast malignancy. Breast cancer is caused by numerous endo- and exogenous risk factors. The paper presents estrogen metabolism, in particular 17β-estradiol and related hormones. The mechanisms of estrogen carcinogenesis include the participation of estrogen receptors, the genotoxic effect of the estrogen metabolites, and epigenetic processes that are also presented. The role of reactive oxygen species in breast cancer has been described. It called attention to a role of numerous signaling pathways in neoplastic transformation. Chemoprotective agents, besides other phytoestrogens, classical antioxidants, synthetic compounds, and their mechanisms of action have been shown.

## Introduction

Breast cancer is the most common malignancy in women and the second leading cause of cancer-related deaths in developed industrialized countries. In 2020, there were an estimated 2.261 million new cases (11.7% of all sites) and 0.685 million new deaths (6.9% of all sites) from breast cancer affecting women worldwide [1]. In Poland breast cancer is responsible for about a quarter of all cancer cases (in 2010 over 15,700 cases). It was assessed that within the next 15 years the number of new cases will exceed 21, 000 and the risk of breast cancer will be comparable to that observed in Europe (roughly 90/10). In Poland, the mortality level from breast cancer is alarming. The countries with 1.5–2 times higher incidence of breast cancer than Poland have identical levels of mortality [2].

Breast cancer is a complex disease. It is one of most malignant cancers with an increased risk of relapse and metastasis. No single factor is likely to be the cause of it. Many endogenous and exogenous factors have been identified with relevance to breast cancer etiology. Age is the strongest risk factor for this disease. More than 2/3 of all new cases occur after the age of 55 and women older than 65 have a relatively greater risk than 4.0 in comparison with those younger than 65. Many additional risk factors for breast cancer have been identified. Some risk factors are invariable, such as age, BRCA1and BRCA2 gene mutations, family history, and reproductive history. Others are potentially variable, such as high endogenous estrogens, hormone therapy, obesity and alcohol consumption. These endogenous factors include increasing age, menarche age (the younger the age during the first menarche, the higher the risk), and menopause age (the older the age at menopause, the higher the risk) [3, 4].

Moreover, there are exogenous risk factors known as external and environmental factors. Among others these comprise: alcohol consumption, poor diet, contraceptive pill and hormone replacement therapy, overweight and obesity, sleep deprivation, and physical inactivity [5–7].

Prolonged lactation and physical activity can decrease the number of ovulatory cycles. A meta-analysis revealed that women could significantly reduce the risk of breast cancer by approximately 25% if they exercise regularly. The interdependence was most evident in post-menopausal women who performed moderate to vigorous exercises throughout their lifetime [8]. Physical activity leads to lower circulating steroid hormone levels, mainly estrogens, and other key hormones, such as leptin and insulin. Physical activity reduces levels of chronic inflammation factors, e.g., C-reactive protein (CRP), which can contribute to breast cancer risk. Physical activity also activates immune system surveillance and decreases oxidative stress [9].

It has been suggested that dietary and environmental factors may be responsible for up to 50% of breast cancer incidence [10]. A low-fat diet and a vegetarian diet [11] decrease levels of sex-steroid hormones. These data suggest that reduction of serum sex-steroid hormone concentrations may diminish the risk of breast cancer.

The normal physiological growth, proliferation and differentiation of breast tissues as well as the development and progression of breast malignancy [12] are mediated by 17β-estradiol (E_2_) and related compounds upon binding to two members of the nuclear receptor superfamily—estrogen receptor (ER) ERα and ERβ [13].

Upon ligand binding, ERs undergo a conformational change which allows chromatin interaction and the regulation of target genes transcription [14].

Both estrogens and androgens play critical roles in the development of breast cancer. Higher levels of serum estradiol and estrone (E_1_) were found in post-menopausal women with breast cancer compared to controls [15]. In a case–control study which included postmenopausal women, a positive association with serum E_1_, androstenedione, and inverse association with sex hormone-binding globulin (SHBG) after adjustment for hormonal variables and body mass index (BMI) were observed [16]. In another study, free E_2_, albumin-bound estradiol and estrone were all associated with an increased risk of breast cancer after adjustments of BMI in a prospective study [17]. There are data that post-menopausal women with breast cancer also show high serum levels of other sex hormones, i.e. testosterone, dehydro-epiandrosterone sulfate (DHEAS) and androstendione. These hormones are precursors of estrogenes [18]. Estradiol is biosynthesized from androstendione via testosterone or E_1_ [19]. In a case–control study which included white women with breast cancer and proper controls, the age-adjusted relative risk (RR) of breast cancer in women with the highest level of E_2_ and free testosterone was 3.6 (95% confidence interval, CI 1.3–10.0) and 3.3 (95% CI 1.1–10.3), respectively, in comparison with those with the lowest concentrations of both hormones [20].

In a prospective case–control study conducted on women with invasive breast cancer and control subjects aged 55–74 years, the serum concentration of unconjugated E_2_ was strongly associated with the risk of breast cancer (hazard ratio, HR = 2.07; 95% CI 1.19–3.62) [21]. In addition, an enhanced 2-hydroxilation of estrogens, E_1_ and E_2_, was associated with reduced risk of postmenopausal breast cancer [22]. There is a suggestion that more extensive 2-hydroxylation of estrogens is associated with lower risk, whereas less extensive methylation of 4-hydroxylated catechols is associated with higher risk of postmenopausal breast cancer [21].

Relatively few studies have reported estrogen and breast cancer risk in pre-menopausal women. One research suggested that mean plasma estradiol concentrations may be higher in pre-menopausal women than in controls but were found not statistically significant in either of these studies [23]. Another study conducted on premenopausal breast cancer women and suitable controls demonstrated that higher urinary estrone (E_1_) and E_2_ levels were significantly associated with lower risk of breast cancer. Also, inverse association with parent estrogen metabolites and the parent estrogen metabolite/non-parent estrogen metabolite ratio were observed and it has been suggested that these women are at lower risk [24].

## Metabolism of endogenous estrogens

**Fig. 1:**
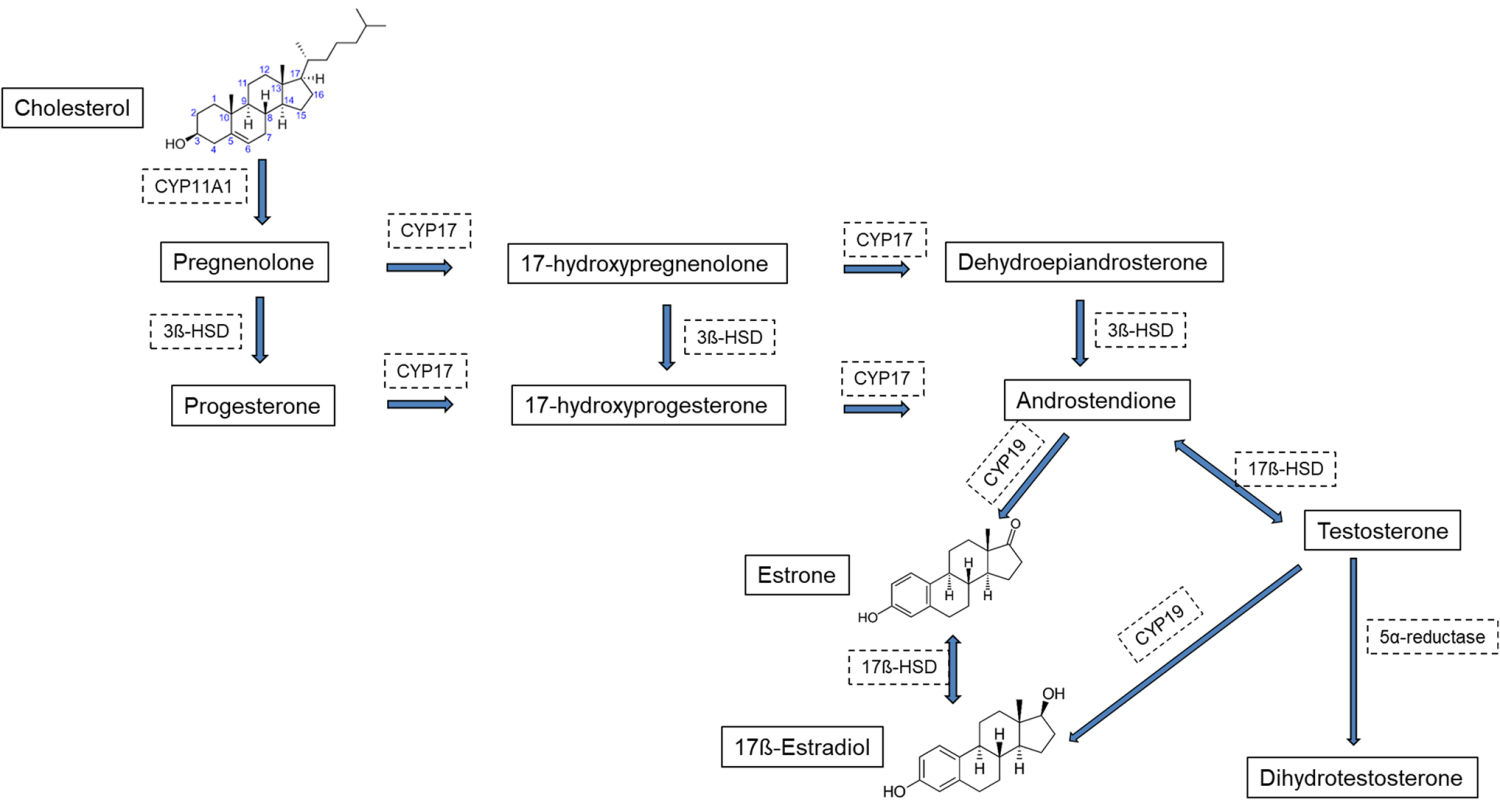
Synthesis of sex hormones. *CYP11A1* mitochondrial cholesterol side-chain cleavage enzyme, P450_scc_, *3β-HSD* 3β-hydroxysteroid dehydrogenase, *CYP17* 17α-hydroxylase/17,20-lyase, P450_c17_, *CYP19* aromatase, P450_aro_, *17β-HSD* 17β-hydroxysteroid dehydrogenases

Estrogens are synthesized by the ovaries and partly by the adrenal gland in the woman’s body. Cholesterol is a precursor of all steroid hormones (Fig. 1). The main source of this compound is LDL-cholesterol [25]. Cholesterol is metabolized to the 21-, 19-, and 18-carbon steroid hormones, respectively [26].

The first step in ovarian steroidogenesis is the passage of cholesterol into the mitochondria. The next step that the biotransformation of cholesterol to pregnenolone, catalyzed by the mitochondrial enzymes. Pregnenolone is a final precursor for everything sex steroid hormones. This compound is biotransformed to progesterone or androstenedione. Androstenedione is transformed to other androgens or estrogens, i.e., testosterone, E_1_, and E_2_ [26].

Testosterone is rapidly demethylated at the C-19 position and converted to E_2_ by the action of steroid aromatase (CYP19) in the peripheral tissues. CYP19 is the rate-limiting enzyme in the conversion of androgens to estrogens. An aromatase mediates in the metabolism of C19 androgen steroids to estrogens. This enzyme and its mRNA have been demonstrated in both the epithelial cells of the terminal ductal lobular units and stromal cells of normal human breast [27]. The high activity of CYP19 may increase breast cancer risk [28] by providing more estrogen for activation to genotoxic metabolites and by stimulating breast epithelial cell mitosis. The blocking CYP19 activity is an important pharmacological method for the treatment of estrogen-dependent breast cancer. The parent estrogens, E_1_ and E_2_, are reversibly inter-converted by the 17β-hydroxysteroid dehydrogenase enzyme. In premenopausal women, E_2_ synthesized in the ovaries is the most important estrogen, whereas in postmenopausal women, E_1_ present in peripheral tissues is predominant [26]. An important source of estrogens is the peripheral conversion of androgens in the adipose tissue to free estrogens.

**Fig. 2:**
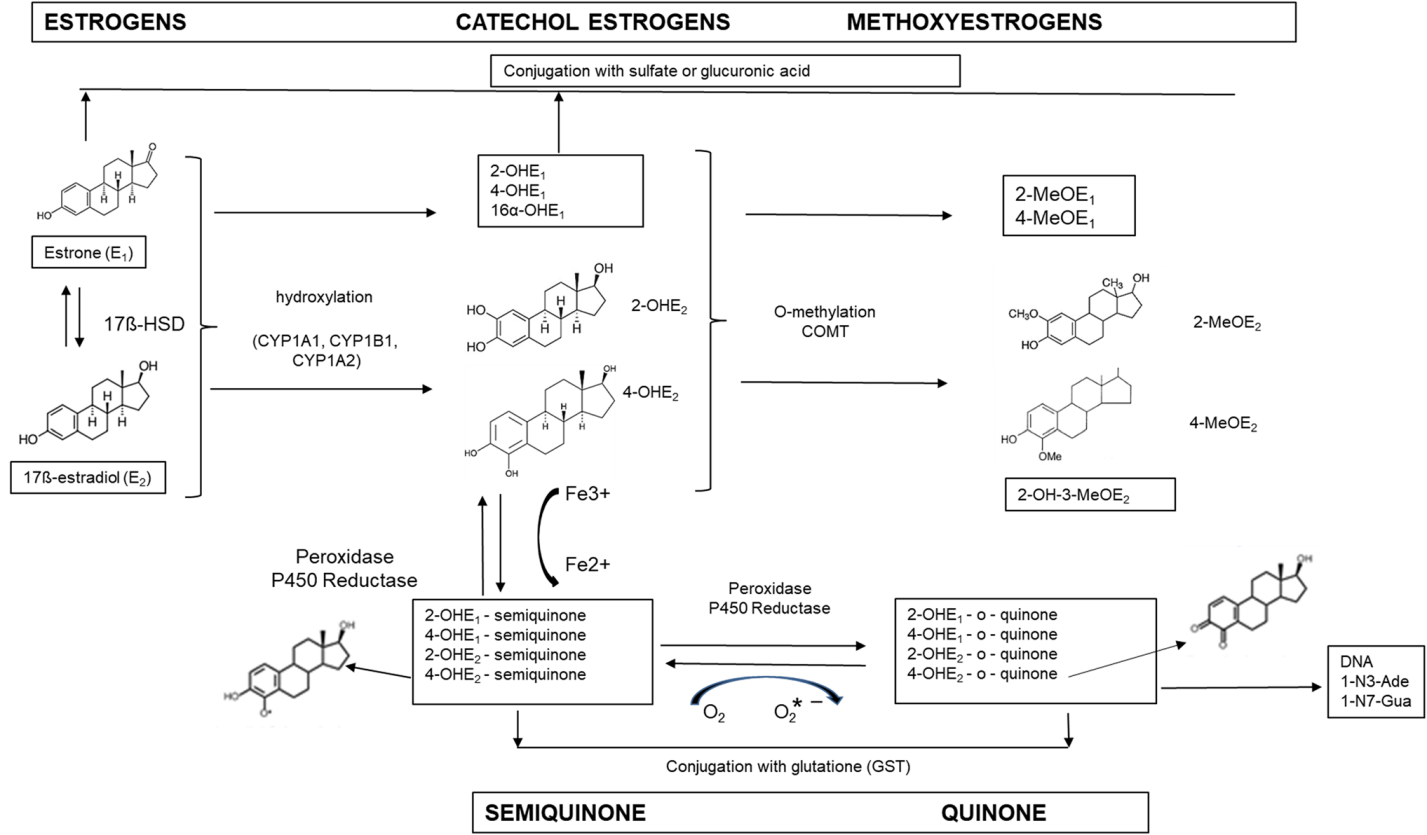
Metabolic pathway of estrogens. *2-OHE*_*1*_ 2-hydroxyestrone, *4-OHE*_*1*_ 4-hydroxyestrone, *16α-OHE*_*1*_ 16α-hydroxyestrone, *2-OHE*_*2*_ 2-hydroxyestradiol, *4-OHE*_*2*_ 4-hydroxyestradiol, E_3_ estriol, *2-MeOE*_*1*_ 2-methoxyestrone, *4-MeOE*_*1*_ 4-methoxyestrone, *2-MeOE*_*2*_ 2-methoxyestradiol, *2-OH-3-MeOE*_*2*_ 2-hydroxy-3-methoxyestradiol, *4-MeOE*_*2*_ 4-methoxyestradiol, *17β-HSD* 17β-hydroxysteroid dehydrogenases, *COMT* catechol–O–methyltransferase, *GST* glutathione S-transferase, *CYP* cytochrome P450, *1N3-Ade* 1–N3–Adenine, *1-N7-Gua* 1–N7–Guanine

Estradiol and estrone are metabolized by three competitive pathways involving irreversible hydroxylations catalyzed by the NADPH-dependent cytochrome P450 (CYP) enzymes including CYP1A1, CYP1B1, and CYP1A2 (Fig. 2). E_1_ and E_2_ are hydroxylated at positions C2, C4 and C16. Therefore, both E_1_ and E_2_ are biotransformed to catechol estrogens including 2-hydroxyestrone (2-OHE_1_), 4-hydroxyestrone (4-OHE_1_), 2-hydroxyestradiol (2-OHE_2_), 4-hydroxyestradiol (4-OHE_2_), 16α-hydroxyestrone (16α-OHE_1_), or estriol. Estriol is produced by the hydroxylation of E_2_ or reduction of 16α-OHE_1_ [26]. Estriol is considered to be a peripheral metabolite and inactivation product of E_1_ and E_2_ because it is biologically less active than estrogens.

Catechol estrogens are methylated by the catechol–O–methyltransferase (COMT) to 2-, 3- and 4-methoxyestrogens. From hydroxyestradiols COMT generates two products, 2-methoxyestradiol (2-MeOE_2_) and 2–hydroxy–3–methoxyestradiol (2–OH–3–MeOE_2_), from 2-OHE_2_, but only one product, 4-methoxyestradiol (4-MeOE_2_), from 4-OHE_2_ [29]. The *O-*methylation of catechol estrogen further blocks their two-electron oxidation to semiquinones (SQ) and quinones (Q). Parent estrogens, catechol estrogens, and methoxyestrogens can be also conjugated with glucuronic acid, sulfate, and glutathione by UDP-glucuronosyltransferases, sulfotransferases (SULTs), and glutathione S-transferase P1 (GSTP1), respectively. These enzymes are expressed in breast tissue. SULTs inactivate estrogens because the introduction of the sulfate group prevents the binding of the steroid to its receptor and reduces its mitogenic effects. GSTP1 yields two conjugates, 2–OHE_2_–1–SG and 2–OHE_2_–4–SG, from the corresponding quinone 2–hydroxyestradiol–quinone and one conjugate, 4–OHE_2_–2–SG, from 4–hydroxyestradiol–quinone [29]. Conjugation is a detoxication process by which estrogens either become water soluble and are excreted in the urine or feces, or turn into a more lipophilic moiety with longer half-lives [30].

When catechol estrogen biosynthesis is increased and/or COMT and other conjugated enzymes activity is decreased, the catechol estrogens are easily oxidized to reactive SQ and Q. These electrophilic products, E_1_/E_2_–3,4–quinones (E_1_/E_2_–3,4–Q), react with DNA to form the depurinating adducts 4–OHE_1_/E_2_–1–N3Adenine (4–OHE_1_/E_2_–1–N3Ade) and 4–OHE_1_/E_2_–1–N7Guanine (4–OHE_1/_E_2_–1–N7Gua), which constitute over 99% of the total DNA adducts formed [31, 32]. These adducts are rapidly lost from DNA by cleavage of the glycosyl bond. The apurinic sites, as a result of oxygenation reaction, generate the mutations that may lead to the initiation of cancer [33, 34].

The 2-hydroxylation process is a major metabolic pathway compared to the 4- and 16-hydroxylation pathways. The CYP1A1/2 and CYP3A4 catalyze C2 hydroxylation of parent hormones to their respective catechol products. The catechol estrogens possess a low binding affinity for the ER [35]. These metabolites show lower hormonal potency when compared to E_2_, and both non-estrogenic and anti-estrogenic activities. There is some evidence that 2-OHE_1_ and 2-OHE_2_ inhibit cell growth and proliferation [36]. In addition, 2-hydroxyestrogens have been associated with normal cell differentiation and apoptosis [37].

The lack of carcinogenic activity of 2-hydroxyestrogens has been attributed to a high rate of clearance, more rapid rate of *O*-methylation and lower hormonal potency in estrogen target tissues, plus produces 2-methoxyestradiol, which suppress tumor cell proliferation and angiogenesis [19]. On the other hand, it has been shown that 2-hydroxyestrogen can damage DNA and generate free radicals when they go through redox cycling or when COMT is inhibited [38]. Methoxyestrogens, including 2-methoxyestradiol (2-MeOE_2_) and 4-methoxyestradiol (4-MeOE_2_), have been shown to inhibit both CYP1A1 and CYP1B1 activities. For both enzymes, the order of inhibition by methoxyestrogens was 2–OH–3–MeOE_2_ > or = 2-MeOE_2_ > 4-MeOE_2_. In the in vitro study it was demonstrated that CYP1A1 and CYP1B1 O-demethylated the methoxyestrogens to catechol derivatives [39]. Thus, both the CYP-mediated oxidation of estrogens and the *O*-demethylation of methoxyestrogens yield identical products, catechol estrogens.

2-Methoxyestradiol exhibits potent apoptotic activity against rapidly growing tumor cells and possesses antiangiogenic action. This metabolite has a lower binding affinity for both ERα and ERβ compared with that of E_2_ [40]. 2-MeOE_2_ has a 500- and 3200-fold lower affinity than that of E_2_ for α and β estrogen receptors, respectively [41]. While 2-OHE_2_ enhanced growth of MCF-7 breast cancer cells, 2-MeOE_2_ inhibited DNA synthesis and mitosis. It was suggested that 2-MeOE_2_ acts as a cytostatic, whereas, 2-OHE_2_ may be the potent mitogen [42].

There are polymorphisms in the genes encoding CYP1A1, CYP1A2, CYP3A4, and CYP1B1. Some products of this gene exhibit altered kinetics with distinctly lower or higher Km values for both 2- and 4-hydroxylation of 17β-estradiol. For example, in the CYP1B1*3 product a lower Km value for both 2- and 4-hydroxylation has been found [43]. In a case–control study, the carriers of the CYP1B1*3 allele were significantly more frequent among breast cancer women, than those in the controls [44].

Enzymes of the CYP3A family are most important in hepatic drug metabolism because of their wide substrate specificity. The CYP3A4 hydroxylates E_1_ to 16α-hydroxyestrone, a metabolite involved in breast cancer induction [45]. CYP3A4 and CYP3A5 mRNAs have been detected in mammary tissues [46].

A genetic polymorphism of COMT, creating a COMT(Val) → COMT(Met) substitution at codon 158, is associated with low enzyme activity. It was suggested that its carriers may be at increased breast cancer risk. In a study of pre-menopausal women, a significantly higher risk for breast cancer with COMT(Met/Met) genotypes, compared with the homozygotes COMT(Val/Val) genotype women, was revealed. Women with high BMI and the COMT (Met/Met) genotype demonstrated high breast cancer risk (odds ratio (OR) = 5.7; 95% CI 1.1–30.1) [47]. In Taiwanese women it was observed that of the three gens, i.e., CYP17, CYP1A1, and COMT, low activity of the COMT genotype was associated with the highest relative risk of breast cancer (RR = 4.0; 95% CI 1.12–19.8) [48].

## The mechanisms of mammary gland carcinogenesis

**Fig. 3:**
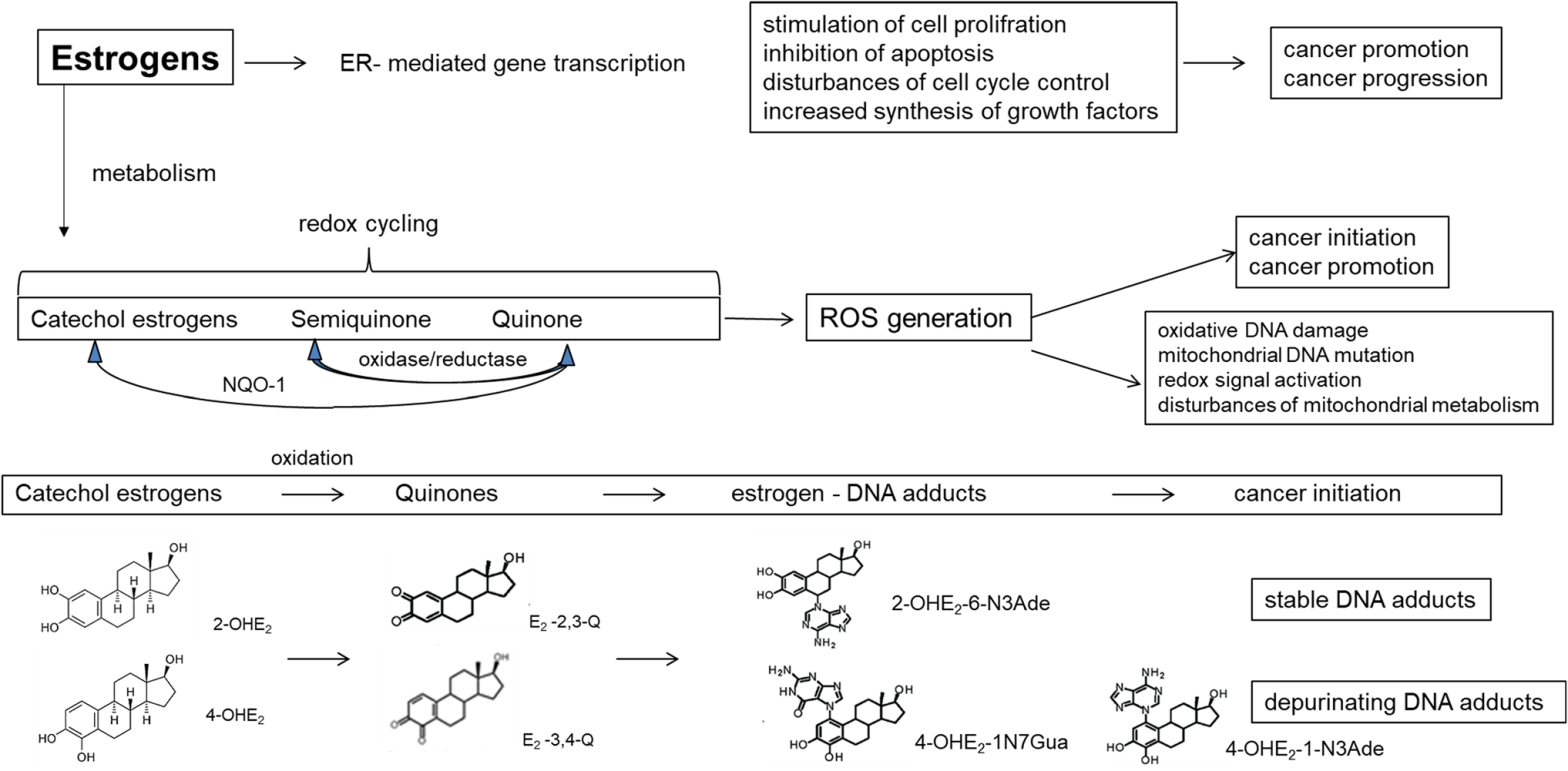
Mechanisms of the carcinogenic effect of estrogens in breast cancer. *NQO-1* NADPH-quinone oxidoreductase 1, ROS-reactive oxygen species, *2-OHE*_*2*_ 2-hydroxyestradiol, *4-OHE*_*2*_ 4-hydroxyestradiol, *E*_*2*_–*2,3*–*Q* estradiol–2,3–quinone, *E*_*2*_–*3,4*–*Q* estradiol–3,4–quinone, *2*–*OHE*_*2*_–*6*–*N3Ade* 2–hydroxyestradiol–6–N3 Adenine, *4*–*OHE*_*2*_–*1N7Gua* 4–hydroxyestradiol–1N7Guanine, *4*–*OHE*_*2*_–*1*–*N3Ade* 4–hydroxyestradiol–1–N3 Adenine

Breast cancer is mainly a disease caused by DNA mutations and epigenetic disorders that make an abnormal cell multiply uncontrollably with the potential to invade other parts of the organism, which leads to its death. Carcinogenesis is usually considered as a process that starts with genotoxic effects—initiation followed by enhanced cell proliferation—promotion. In the breast the main estrogen, E_2_, is both a substrate for the metabolizing enzymes and a ligand for the ER (Fig. 3).

Estrogen carcinogenesis can be connected with ER mediated growth and proliferation as a result of the hormone’s ability to stimulate the expression of genes encoding different growth factors [49]. DNA depurinating adducts formation derived from activated metabolites, e.g., catechol estrogens, semiquinones, quinones and free radicals, generated during estrogen metabolism, and also production of reactive oxygen species (ROS) may play a critical role in breast cancer initiation [49]. Excess ROS not only exerts genotoxicity, but also increases progression of mammmary carcinogenicity by inducing a redox-associated signaling pathway [50].

In experimental conditions, it was demonstrated that MCF-7 breast cancer cells and normal breast tissue in aromatase transfected mice contain the enzymes necessary for the metabolism of E_2_ to the estradiol DNA adducts. After administration of E_2_ to animals the genotoxic products in breast tissue were observed. In ERα knockout (ERKO/Wnt) transgenic animals a reduction in incidence of tumor formation and a delay in the occurrence of those that formed were found. Oophorectomy reduced the incidence of tumors and delayed their onset, whereas E_2_ add-back returned the incidence rate to that observed before oopherectomy. The aromatase inhibitor, letrazole, delayed the onset of tumor formation. These data support both ER-dependent and genotoxic ER-independent effects of E_2_ mediated breast cancer development [51, 52].

The metabolism of estrogens can lead to DNA damage in two ways. The first way is exemplified by the formation of estrogen-adenine/guanine adducts which are released from the DNA by rapid depurination. This phenomenon is a consequence of oxidation of the catechol estrogen metabolites, 4-OHE_1_/E_2_ and 2-OHE_1_/E_2_, to the quinones, i.e., E_1_/E_2_–3,4–Q and E_1_/E_2_–2,3–Q.

The reaction of E_2_–3,4–Q with DNA mainly affords the depurinating adducts 4–OHE_2_–1–N3Ade and 4–OHE_2_–1N7Gua, whereas the reaction of E_2_–2,3–Q with DNA leads to synthesis of the depurinating adduct 2–OHE_2_–6–N3Ade. The E_2_–3,4–Q is much more reactive with DNA than E_2_–2,3–Q. The levels of depurinating adducts formed from E_2_–3,4–Q are much higher than that of the depurinating adduct from E_2_–2,3–Q [52].

Increased levels of estrogen quinones and depurinating adducts occur when estrogen metabolism is unbalanced. This unbalanced metabolism is the result of overexpression of estrogen activating enzymes and/or deficiency of the deactivating enzymes. Treatment of MCF-10F cells, which are ERα-negative and ERβ-positive human breast epithelial cells, with E_2_, 4-OHE_2_ or 2-OHE_2_ induces their neoplastic transformation in vitro, even in the presence of the antiestrogen. This suggests that the transformation process is independent of the estrogen receptor and is more probably due to the genotoxic effect of the estrogen metabolites [53].

The second reason for DNA damage is because of the oxidation of the catechol estrogen metabolites 4-OHE_2_ and 2-OHE_2_ to the quinones, i.e., E_2_–3,4–Q and E_2_–2,3–Q. The quinones, as well as catechol estrogens activated by lactoperoxidase, tyrosinase or prostaglandin H synthase in vitro react with DNA to depurinating and stable adducts which are endogenous initiators of breast cancer. The E_2_–3,4–Q is much more reactive with DNA than E_2_–2,3–Q [54]. Moreover, generation of oxygen-free radicals, e.g., the hydroxyl radical and then hydrogen peroxide, resulting from redox cycling of 4-OHE_2_ to the E_2_–3,4–Q and back conversion to 4-OHE_2_, lead to oxidative modification of DNA. If these processes are sufficient, a number of mutations accumulate over a long period to induce neoplastic transformation.

The catechol estrogens, including 2-OHE_2_, 4-OHE_2_, 2-OHE_1_ and 4-OHE_1_, undergo redox cycling, which results in the production of ROS. These catechol estrogens were found to generate H_2_O_2_ and hydroxyl radicals in lysates of MCF-7, MDA-MB-231 and MCF-10A breast epithelial cells. Intracellular ROS accumulation leads to oxidative DNA modification expressed by 8–oxo–7,8–dihydroxy–2′–deoxyguanosine (8–oxo–dG) level [55]. Dicoumarol, the inhibitor of NAD(P)H:quinone 1 (NQO1) activity, enhances 4-OHE_2_-induced ROS production, whereas, the antioxidant *N*-acetylcysteine inhibites ROS accumulation and the neoplastic transformation induced by 4-OHE_2_ [50]. Redox cycling of the quinones and semiquinones and subsequent generation ROS is an important mechanism of DNA damage and cell death.

In cancerogenesis induced by estrogen and their metabolites activation of the numerous signaling pathways occurs.

A high level of ROS increased the translocation of the nuclear factor—kappa B (NF-kB) to the nucleus and its DNA binding through induction of Ikappa B kinase alpha (IκK alpha) and IκK beta activities. The inhibition of the IκK activities significantly reduced the independent growth colony formation in MCF-10A cells treated with 4-OHE_2_ [50]. The redox-sensitive transcription factor in the nucleus, factor ĸB (NF-ĸB), was transiently activated by 4-OHE_2_ exposure. Co-exposure of MCF-10A cells to the NF-ĸB inhibitor increased cell death induced by 4-OHE_2_. In addition, 4-OHE_2_ caused transient activation of extracellular signal-regulated protein kinases (ERK). An inhibition of ERK aggravated the 4-OHE_2_-induced cytotoxicity. ERK plays the main role in protection against catechol estrogen-induced cell death [56].

ROS, not only leads to oxidative DNA damage, but also promotes neoplastic transformation of initiated cells, which is a critical process in the induction of carcinogenesis. There is a suggestion that 4-OHE_2_-induced cell transformation is independent of ER binding [53]. When MCF-10F cells were exposed to benz[a]pyrene, E_2_, 2-OHE_2_, 4-OHE_2_, or 16α-OHE_2_, 4-OHE_2_ effectively induced larger colonies at low doses [57]. It was observed that the effect of 4-OHE_2_ on neoplastic transformation in MCF-10A cells take places via activation of IκK-NF-ĸB signaling pathways [50].

During the malignant transformation process in MCF-10A cells treated with 4-OHE_2_ the increased AKT phosphorylation through phosphatidylinositol 3-kinase (PI3K) activation was observed. These data indicate that 4-OHE_2_-induced cell transformation may be mediated through redox-sensitive serine/threonine protein kinase (Akt) signal transduction pathways by up-regulating the expression of cell cycle genes CDC2, PRC1, and PCNA, and also the transcription of nuclear respiratory factor-1 (NRF-1). The study has demonstrated that 4-OHE_2_ is the main estrogen metabolite responsible for induction of malignant phenotype cells. Moreover, ROS are crucial species in the development of estrogen-induced breast cancer [57].

4-OHE_2_ not only induces cell transformation but also promotes the growth of cancer cells. There are a lot of studies that 4-OHE_2_ stimulated specific intracellular signaling pathways, which supports the role of 4-OHE_2_ in oncogenesis. In particular, the effect of E_2_ metabolites on the cell cycle has been investigated [58]. Among these metabolites, 4-OHE_2_ and 16-OHE_1_ showed a proliferative effect on MCF-7 human ER-positive metastatic breast cancer cells, which was accompanied by a downregulation of apoptosis [59]. On the other hand, the expression of cell cycle-related genes such as CDC2, the protein regulator of cytokinesis 1 (PRC1), the proliferation of the cell nuclear antigen (PCNA), and the transcription factor NRF-1 were up-regulated by 4-OHE_2_ [57].

It has been demonstrated that 4-OHE_2_ induces hypoxia-inducible factor-1 alpha (HIF-1) and vascular endothelial growth factor A (VEGF-A) expression in two human ovarian cancer cell lines, OVCAR-3 and A2780-CP70 cells, in dose- and time-dependent manners. HIF-1 is a transcription factor which is induced by hypoxia, growth factors, and activation of oncogens. HIF-1 activates downstream target genes such as VEGF-A, which plays a significant role in tumor progression and angiogenesis. Moreover, it was observed that PI3K inhibitors blocked HIF-1 alpha and VEGF-A expression, whereas the mitogen-activating protein kinase (MAPK) inhibitor did not alter the expression of both indices induced by 4-OHE_2_. 4-OHE_2_ also induced AKT phosphorylation at Ser473. These data suggest that the PI3K/AKT/FRAP signaling pathway is required for HIF-1 alpha and VEGF-A expression induced by 4-OHE_2_, although the MAPK pathway is not required. The findings that induction of HIF-1 alpha and VEGF-A expression occurs via the activation of the PI3K/AKT/FRAP signaling pathway could be an important mechanism of 4-OHE_2_-induced tumorogenesis [60].

In MCF-7 and MCF-10A CYP1B1 promoted cell proliferation, migration, and invasion. CYP1B1 induced epithelial–mesenchymal transition (EMT) and activated Wnt/β-catenin signaling pathway via upregulation of a key oncogenic proteins. Specific protein 1 (Sp 1), a transcription factor involved in cell growth and metastasis is upregulated by CYP1B1. The suppression of Sp 1 expression by mithramycin A blocked oncogenic transformation induced by CYP1B1.This indicates that Sp 1 acts as a key mediator for CYP1B1 action. Treatment of MCF-7 and MCF-10A cells with 4-OHE_2_ showed similar effects to the CYP1B1 overexpression. These data suggest that both CYP1B1 and 4-OHE_2_ promote cell proliferation and metastasis by inducing EMT and Wnt/β-catenin signaling via Sp 1 induction [61]. In addition, CYP1B1 promotes cancer cell survival through involvement of DNA hypermethylation-mediated death receptor 4 (DR4) inhibition via Sp 1, which may act as a key mediator for oncogenic effect [62].

The invasion and metastases of cancer follow the degradation of the extracellular matrix, which acts as a barrier to cancer cells. The proteins of the extracellular matrix are degradated by proteases, mainly the matrix metalloproteinases (MMPs), which increase the risk of invasive and metastatic processes. It was demonstrated that 4-OHE_2_ led to the conversion of pro MMP-2 and pro MMP-9 to activate both MMPs, which resulted in dissemination of cancer cells [63].

## Chemoprevention

Chemoprevention alone and chemoprevention in combination with anticancer treatment is an important way to reduce morbidity and mortality caused by breast cancer [64].

Numerous chemical agents, natural and synthetic, exert a protective effect on the breast cancer cells. These agents include, among others, phytoestrogens, aromatase inhibitors, marijuana smoke condensate, classical antioxidants, and synthetic compounds.

There is a theory which postulates that estrogens increase the rate of cell proliferation by stimulating ER-mediating transcription, thereby increasing the number of errors occuring during DNA replication. The other theory suggests that E_2_ is metabolized to Q-derivatives, which directly remove base pairs from DNA through a depurination process. Error-prone DNA repair leads to point mutations. It was postulated that both processes interact in an additive or synergistic manner. Thus, antiestrogens would only block receptor-mediated effects, whereas aromatase inhibitors would inhibit both processes. Therefore, aromatase inhibitors would be more powerful in prevention of breast cancer than antiestrogens. In in vitro studies it was observed that catechol-estrogen metabolites are produced in MCF-7 human breast cancer cells. The animal study obtained the dissociation of ER-mediated function from the effects of E_2_ metabolites. The absence of E_2_ markedly reduced the incidence of tumors and delayed their onset. The metabolites of E_2_ may induce breast cancer simultaneously with the ER-mediated mechanism. The study supported the possibility that aromatase inhibitors might be more effective than tamoxifen and other antiestrogens in prevention of breast cancer [65].

In breast cancer epithelial cells, the mitogenic activity of E_2_ is transduced through binding to two receptors, ERα and ERβ, as transcription factors. Anti-estrogens and aromatase inhibitors are applied in clinic to arrest the estrogen-dependent growth of breast cancer cells. Estrogen effects are mediated through nuclear ERs and cytoplasmic/membrane ERs and G-protein-coupled ERs. These estrogen-binding systems connected with different proteins that lead to cell cycle signaling, proliferation and survival. The partners of nuclear ER include steroid receptor coactivator 1–3 (SRC1-3), histone deacetylases (HDACs), ERβ, and also proteins such as LKB1, PELP1, PAX2 and TOXA1. The extra-nuclear ERα factors include PI3K, and the sarcoma virus tyrosine kinase (Src). These factors are all potential targets for therapeutic agents. In addition, proliferation of breast cancer is enhanced by insulin-like growth factor 1 (IGF-1) and epidermal growth factor (EGF), which stimulate signaling through the MAPK and PI3K/AKT pathways by activation of IGF-1R and EGFR receptors, respectively. These pathways are linked with ER-activated signaling, and the membrane ERα forms complexes with Src and PI3K. Inhibiting these pathways with specific inhibitors or activators may be therapeutically beneficial for arresting breast cancer cell cycle progression and for inducing apoptosis [66]

Phytoestrogens are a biologically active organic compounds derived from plants and have structures like to the main mammalian estrogen, E_2_. There are five major classes of phytoestrogens, namely: flovonoids, isoflavonoids, lignants, coumestans, and stilbenes. The data about association between breast cancer and phytoestrogens issue from epidemiological studies showing that the incidence of breast cancer is low in Asian women who consume high dietary levels of soy products which have a high isoflavone content [67, 68].

Resveratrol, quercetin, daidzein, and genistein represent a group of the most commonly consumed phytoestrogens. In East Asia, the average daily consumption of phytoestrogens is evaluated to be 20–50 mg. In Europe the average daily phytoestrogen consumption is assessed to be 0.49–1.0 mg. Plasma isoflavone concentrations range from 2 µM to 5 nM. However, local tissue phytoestrogen concentrations are probable to be 2–3 times higher than plasma concentrations [67, 68].

Phytoestrogens are thought to exert anticancer actions through different molecular pathways. They act within target cells on estrogen receptors, cell signaling pathways, the cell cycle, apoptosis, steroid synthesis and induction of DNA damage in cancer cells [68].

Numerous studies in humans have examined the effects of phytoestrogens on estrogen biosynthesis and their biosynthetic enzyme activities. It has been observed a decrease of plasma/serum levels of the follicle-stimulating hormone (FSH), luteinizing hormone (LH), E_2_, E_1_, end progesterone following phytoestrogen consumption. In addition, the isoflavone diet increased the ratio of 2-hydroxyestrone to 16α-hydroxyestrone and decreased in the ratio of genotoxic estrogens to total estrogens. In women consuming 40 mg isoflavones every day for 3 months, the average menstruation length and follicular phase of the menstrual cycle were elongated. Flavones and flavonones, including 7-hydroxyflovone, apigenin, chrisin and hesperetin, are the most potent inhibitors of aromatase. Also, quercetin, genistein, and daidzein inhibited the transcription of CYP19 mRNA in human granulosa luteal cells. A mixture of genistein, daidzein, and biochain significantly reduced aromatase activity. Resveratrol exerted inhibitory effects on aromatase activity in MCF-7 cells in vitro and suppressed transcription of CYP19 mRNA in SK-BR-3 breast cancer cells [67].

In addition, phytoestrogens have been shown to inhibit 17β-hydroxyestradiol dehydrogenase; e.g., genistein decreased this enzyme activity in human placental microsomes, granulosa luteal cells, MCF-7, and T47D human breast cancer cells [67]. Phytoestrogens can bind weakly to ERs and some have a preferential affinity for ERβ, which can inhibit the transcriptional growth-promoting activity of ERα. Instead, only higher doses of phytoestrogens exert their growth inhibitory effects through the ERα and Erβ. It has also been shown to inhibit oncogenic cyclin D1 expression, but increase the level of cyclin-dependent kinase inhibitors, p21 and p27, and tumor suppressor genes p53, APC, AMP, and mammary serpin peptidase inhibitor (SERPINB5). These effects were only observed at high doses (> 10 µmol/l) of phytoestrogens. The effects of phytoestrogens on breast cancer may be also caused by their ability to inhibit local estrogen synthesis, induce epigenetic changes, and suppression of ERα signaling pathways [68, 69].

The majority of breast cancer patients have ER-positive breast cancer. E_2_ is the most potent endogenous estrogen, which expresses its activity through binding to ERs. Most of the growth-inducing effects of estrogens are exerted by their link to ERα. ERα is considered the first target for inhibition of tumor proliferation. In contrast, the proliferative activity of estrogens can be opposed by ERβ. In breast cancer cells E_2_ induces proliferation and inhibits apoptosis via ERα. ERβ inhibits the growth effects of ERα. ERβ-mediated inhibitory effect against ERα depends on the participation of transcription factors able to repress most of the genes activated by ERα and on the degradation of this receptor [70].

### Furanodienone

The ERα is critical in the development and progression of breast cancer. The mitigation of ERα activities is a promising strategy in pharmacotherapy. Furanodienone is the main bioactive compound of *Rhizoma Curcumae.* It is commonly used in Chinese medicine for cancer therapy. This compound in vitro inhibited MCF-7, T47D, and MDA-MB-231 breast cancer cells’ proliferation in a dose-dependent manner. ERα-negative MDA-MB-231 cells were less sensitive to furanodienone than ERα-positive MCF-7 and T47D cells. Furanodienone effectively blocked E_2_-stimulated MCF-7 cell proliferation and cell cycle progression, and induced apoptosis. Furanodienone down-regulated ERα protein and mRNA expression levels without effect to ERβ expression. Exposure to furanodienone inhibited E_2_-stimulation of the estrogen response element (ERE) activity and oblated E_2_-targeted gene, e.g., oncogenic transcription factor (c-Myc), B-cell lymphoma-2 (Bcl-2), and cyclin D1 expression, which manifested by the inhibition of the cell cycle and cell proliferation and also induction apoptosis. Diminished the cell growth inhibitory effect of furanodienone in MCF-7 cells with knockdown of ERα suggest that these effects are mediated by inhibition of ERα signaling pathways [71].

Basal phenotype breast cancer is one of the most aggressive tumors that frequently metastasizes to the brain.

Current antihormonal therapies are frequently applied for the treatment of hormone receptor positive breast cancer, i.e., ERα and/or nuclear progesterone receptors (PR-positive). For ERα breast cancers, antiestrogen therapies, such as tamoxifen and anastrozole, are often efficient both in primary and in metastatic stages. The status of PR expression is used with ER to indicate the potential effectiveness of antiestrogen therapies because the majority of breast cancers express ER and PR concurrently. Clinical studies revealed that ERα/PR-negative breast cancers respond less well to selective ER modulator (SERM) therapy than ERα/PR-positive tumors [72, 73]. Recent data suggest that ERα/PR-negative breast cancers may be resistant to SERM, whereas they may be less resistant to estrogen withdrawal therapy with aromatase inhibitors. Novel molecular mechanisms explaining SERM resistance in ERα/PR-negative tumors have suggested that growth factors may downregulate PR levels. The absence of PR may reflect hyperactive cross talk between ER and growth factor signaling pathways that downregulate PR even as they activate other ER functions. It was concluded that ERα/PR-negative breast cancers might be treated by blocking ER activity via estrogen withdrawal with aromatase inhibitors by targeted ER degradation, or by combined therapy that assumes both ER and growth factor signaling pathway [73].

### Marijuana smoke condensate

There are data about antiestrogenic effects of marijuana smoke condensate (MSC) and cannabinoid biological active compounds. MSC, but not Δ9-tetrahydrocannabinol, cannabidiol, and cannabinol, induced the antiestrogenic effect via the ER-mediated pathway.

Moreover, MSC induced CYP1A1 activity and the E_2_ metabolism, but inhibited the aromatase activity, suggesting that the activity of MSC is also associated with the indirect ER-dependent pathway as a result of the depletion of the E_2_ level available to bind to the ER. It was suggested that pyrogenic products, including polycyclic aromatic hydrocarbons (PAHs), might be responsible for the antiestrogenic activity [74].

### Genistein

An isoflavon, genistein occurs in soybeans. Cell proliferation and apoptosis inhibition in MCF-7 (high ratio of ERα/ERβ), although not in T47D (low ratio) and MDA-MB-231 (ER-negative), cells were observed after E_2_ and genistein treatment simultaneously. Moreover, exposure to genistein produced an up-regulation of ERβ and an increase in cytochrome c oxidase activity in T47D cells and decreased the ATP synthase/cytochrome c oxidase ratio. The beneficial effects of genistein treatment depend on the ERα/ERβ ratio in breast cancer cells. Genistein treatment leads to cell cycle arrest and an improvement of mitochondrial function in T47D cells with a low ERα/ERβ ratio, but not MCF-7 with a high ERα/ERβ ratio and MDA-MB-231 with ER-negative ones [75]. In another study genistein treatment of breast cancer cells in combination with cytotoxic agents, *i.e*., cisplatin, paclitaxel or tamoxifen, caused an increase in cell viability, and also in the antioxidant enzymes level, and decreased ROS production, autophagy, and apoptosis, plus enhanced the cell cycle S phase only in MCF-7 cells with a high ERα/ERβ ratio. In contrast, in the T47D (low ratio) and overexpressed ERβ cells the combination of genistein with cytotoxic compounds did not cause significant changes in the analyzed parameters. It was concluded that genistein consumption is beneficial in patients receiving anticancer therapy with a high ERα/ERβ ratio of breast cancer cells. Also, in the patients with lower ERα/ERβ ratio breast cancer cells genistein consumption could be harmless [76]. Genistein has inhibited invasion of breast cancer cells and decreased tumor growth in nude mice bearing MCF-7 and MDA-MB-231 xenografts.

Genistein, glycitein, daidzein, equol, O-desmethylangolensin and coumestrol exerted a potent inhibitory effect on cell invasion. In contrast, the lignants exerted minimal effects on invasion. Inhibition of invasion induced affects by the phytochemicals was seen without affecting cell viability [77].

Genistein induces apoptosis in breast cancer cells that are ER-positive and ER-negative. Ability of genistein to promote apoptosis requires wild-type caspase-3 activity. Moreover, it was demonstrated that genistein induced apoptosis in breast cancer cells by reducing expression of an oncogenic micro-RNA (miR-155) that occurs in breast cancer. These data indicate that genistein growth inhibitory effects proceed via activation of apoptotic pathways and also suggest that genistein can exert its antitumor actions via estrogen-independent signaling pathways. Genistein, when co-administered with adriamycin, was found to induce necrotic-like cell death in breast cancer cells by inactivating the HER-2 receptor and Akt. It was shown that genistein might be a valuable treatment option for tamoxifen-resistant breast cancer. Inhibition of invasiveness and metastatic activity of MCF-7 and MDA-MB-231 cells by genistein probably proceeds by downregulation of the transcription of various MMPs. Also, many of the anticancer properties of genistein are believed to proceed through its regulation of various molecular signaling pathways that involve numerous genes, such as Bc1-2, Bax, NF-kB, and Akt. There are data that genistein exposure results in the upregulation of BRCA1/2 genes in a dose- and time-dependent manner. Furthermore, genistein upregulates the expression p53 and TNFα genes in breast cancer cells. It was reported that genistein-mediated downregulation of miR-155 contributions to the anticancer effects of genistein in metastatic breast cancer [78].

### Calycosin

The endogenous factors which the increased expression stimulates in the development of breast cancer are as follows: IGF1, PI-9, ERα, BRF2, cyclin D1, pS2, TGF-β3, monoamine oxidase A, and α-antichymotripsin. In contrast, the decreased expression of Akt, Wnt/β-catenin, miR-155, MMPs, and HSD leads to inhibition of tumor proliferation. The increased apoptosis is associated with NF-kB decrease, and p53, Caspase 3, Bax, TNF-α, and BRCA-1/2 elevated expression [78].

Calycosin is an isoflavone derived from the Chinese medical herb *Radix astragoli*. Calycosin at low concentrations effectively stimulated proliferation of ER-positive MCF-7 human breast cancer cells. On the other hand, this isoflavone at high concentrations significantly suppressed the proliferation of these cells and promoted cell apoptosis through the mitochondrial apoptotic pathway by upregulating RASD1. RASD1 is a Ras-family member and a regulator in MAPK-mediated cell proliferation or apoptosis. Calycosin decreased the expression of Bcl-2 and increased Bax expression, which was significantly correlated with elevated RASD1 expression [79]. The cellular effects of isoflavones, i.e., impaired proliferation and triggered apoptosis, induced by calycosin and formononetin were observed only in ER-positive breast cancer cells. No such effects were observed in ER-negative breast cancer cells, indicating the association between isoflavones’ induced inhibitory effect and ERs. After the treatment of MCF-7 cells with calycosin the ERβ expression was significantly increased, followed by a decrease of IGF-1R, activation of poly (ADP)-ribose) polymerase 1 (PARP-1) and downregulation of miR-375. Thus, calycosin exerts an inhibitory effect on breast cancer growth by ERβ-mediated regulation of IGF-1R signaling pathways [80, 81].

In addition, higher doses of calycosin (50, 100, and 150 µM) were found to inhibit migration and invasion of the two cell lines in a dose-dependent manner. Calycosin in a higher dose significantly reduced the expression levels of the forkhead box P3 (Foxp3), followed by downregulation of VEGF and MMP-9 in MCF-7 and T47D breast cancer cells. Thus, a higher dose of calycosin led to a reduction of migration and invasion of human breast cancer cells, by targeting Foxp3-mediated VEGF and MMP-9 expression [82].

The two main isoflavones, calycosin and genistein, inhibited proliferation and induced apoptosis in MCF-7 breast cancer cells. MCF-7 cells’ exposure to calycosin or genistein led to decreased phosphorylation of Akt, and reduced expression of its downstream target, HOTAIR. HOTAIR is the HOX transcript antisense RNA gene located on chromosome 12 and encodes the long non-coding RNAs molecule. HOTAIR is over-expressed in breast cancer. High expression of HATOIR in breast cancer is a predictor of metastasis and poor outcome.

Calycosin is more effective in inhibiting breast cancer growth in comparison with genistein, through its regulation of Akt signaling pathways and HOTAIR expression [83].

### Formononetin

Formononetin is one of the main phytoestrogens of red clover plants. This compound inhibited the proliferation of MCF-7 cells and effectively induced cell cycle arrest. Formononetin downregulated the levels of p-IGF-1R, p-Akt, cyclin D1 protein, and cyclin D1 mRNA expression. Formononetin prevented the tumor growth of human breast cancer cells in nude mouse xenografts. It was found that this phytoestrogen causes cell arrest at the G0/G1 phase by inhibiting of IGF-1/IGF-1R-PI3K/Akt pathways and decreasing cyclin D1 mRNA and protein expression [84].

In a study of the molecular mechanisms involved in the induced apoptosis and effect of the proliferation of ER-positive only, MCF-7 and T47D cells were observed. Formononetin activated the mitogen-activated protein kinase (MAPK) signaling pathway in a dose-dependent manner, which resulted in the increased Bax/Bcl-2 ratio and induced apoptosis in MCF-7 cells. Moreover, when MCF-7 cells were pretreated with a p38MAPK inhibitor before phytoestrogen, apoptosis induced by formononetin was less pronounced. These data indicate that the apoptosis induced by formononetin in human breast cancer cells were associated with the Ras-p38MAPK pathway [85].

### Baicalein

Baicalein (3,4,5-trihydroxyfurane) is the compound from the root of *Scutellaria baicalensis*. It has a broad spectrum of activity, including induction of tumor cell apoptosis, cell cycle arrest, inhibiting tumor proliferation and angiogenesis, and also scavenging free radicals. An in vivo study showed that baicalein inhibited metastasis in the xenograft nude mouse model. Baicalein significantly decreased the expression of AT-rich sequence-binding protein-1 (SATB1) and enhanced the expression of E-codherin. SATB1 is a speciffically expressed matrix attached to regions (MARs) binding protein.

In addition, baicalein downregulated the expression of Wnt1 and β-catenin proteins and the transcription level of Wnt/β-catenin-targeted genes. It was suggested that baicalein has the potential to suppress breast cancer metastasis, probably by inhibition of EMT, which may be attributed to downregulation of both SATB1 and the Wnt/β-catenin [86, 87].

SATB1 plays an important role in the initiation and development of cancer. It regulates the malignant progression of breast cancer. Activation of SATB1 is indispensable for tumor metastasis, for the expression of various genes involved in the growth, proliferation, angiogenesis, invasion, and metastasis.

The Wnt/β-catenin pathway is one of the most common signaling abnormalities occurring in human malignancies [88]. It is estimated that metastasis accounts for 90% of cancer-related mortality [89].

### Luteolin

Luteolin (3′,4′,5,7-tetrahydroxyflavone) is a flavonoid which exhibits antioxidant, anti-inflammatory, antibacterial, antidiabetic and antiproliferative properties. It has been shown that luteolin may inhibit cell proliferation, metastasis, and angiogenesis different types of cancers, including breast cancer. These effects proceed through cell cycle arrest, and apoptosis, and by modulating cell signaling pathways. Luteolin was shown to effectively inhibit the IGF-1 stimulated ERα-positve MCF-7 cell proliferation in a dose- and time-dependent manner, and to suppress ERα-negative MDA-MB-231 cells. Supplementation by luteolin at 0.01% or 0.05% significantly reduced the tumor in nude mice inoculated with MDA-MB-231 cells. It has been shown that this flavonoid significantly inhibits the cell cycle of MCF-7 and MDA-MB-231 cells at the G2/M and S stages. Luteolin can efficiently suppress the migration and invasion of MCF-7 and MDA-MB-231 cells. Luteolin exerts its biological actions through multiple signaling pathways, such as PI3K/Akt, MAPK/ERK1/2, EGFR, and NF-kB [90, 91].

### Benzanilides and dithiobenzanilides

There are synthetic estrogenic receptor modulators. Benzanilides and dithiobenzanilides are novel promising compounds which show selective antiproliferative activity against estrogen-dependent MCF-7 breast adenocarcinoma. Their estrogenic activity was confirmed in MCF-7 transfected with an estrogen receptor reporter plasmid and in HEK 239 cells over-expressing the ERα. Docking studies have shown that one of tested compounds interacts with the receptor in the same cavity as E_2_ [92].

### Resveratrol and its analogs

Natural polyphenols are considered chemopreventive agents able to prevent tumor initiation caused by ROS targeting not only lipids and proteins, but also DNA and RNA in living cells. Free radicals react with phenolic compounds much faster than with lipids or DNA. Therefore, polyphenols protect lipids and DNA from oxidative damage induced by free radicals.

The resveratrol (3,4′,5-*trans-*trihydroxystilbene) present in grapes possesses chemopreventive and chemotherapeutic activities. Although resveratrol shows phytoestrogenic activity, it inhibits the growth of both hormone-dependent and hormone-independent breast cancer cells [93, 94]. This compound inhibited transformation, migration and growth of MCF-10A human breast epithelial cells treated with 4-OHE_2_. Resveratrol treatment suppressed the 4-OHE_2_ induced activation of IκKβ and phosphorylation of IκKα, and consequently NF-κB DNA binding activity and cyclooxygenase-2 (COX-2) expression. Resveratrol suppressed ROS production and phosphorylation of Akt and ERK induced by 4-OHE_2_ treatment. Thus, resveratrol blocks activation of IκKβ-NF-κB signaling pathway and induction of COX-2 expression in 4-OHE_2_-treated MCF-10A cells. These effects lead to suppression of migration and transformation of MCF-10A cells [95].

Resveratrol is extensively metabolized by hormone-dependent ZR-75-1 cells, but only marginally by hormone-independent MDA-MB-231 cells exclusively forming resveratrol–3–*O*–sulfate. Resveratrol–3–*O*-sulfate concentration in the cytoplasm and culture medium was several times higher in ZR-75-1 cells than in MDA-MB-231 cells. The expression of SULT1A1 mRNA showed a close association with resveratrol–3–sulfate formation. It was concluded that SULT1A1-based biotransformation diminishes the anticancer activity of resveratrol in breast cancer cells, which must be considered in patients following oral uptake of this polyphenol as a chemopreventive chemical [96].

It was shown that introduction of additional hydroxyl groups into the stilbene structure increases the biological activity of resveratrol. The 3,3′,4,4′,5,5′-hexahydroxystilbene caused the activation of caspase-8 in MDA-MB-231 cells, whereas, the activation of caspase-9 and caspase-3 was evident in ZR-75-1, MDA-MB-231 and T47D breast cancer cells. Activation of caspase-9 and caspase-3 was associated with a reduction of mitochondrial potential and an increase of p53, which could have been connected with the downregulation of mitochondrial superoxide dismutase (MnSOD). MnSOD is a key enzyme assuring antioxidative defense in mitochondria, the cellular center of ROS generation which can cause a significant reduction of antioxidant defense in cancer cells. An increase of oxidative stress was confirmed by loss of reduced glutathione in tested cells. These data suggest that ROS generating compounds may cause p-53-mediated MnSOD activity reduction and lead to induction p53 transcriptional functions, which subsequently lead to the activation of mitochondrial apoptotic processes. It was suggested that increased ROS generation caused by 3,3′,4,4′,5,5′-hexahydroxystilbene, as a result of the interaction of this chemical with the mitochondrial respiratory chain and a decrease in antioxidative defense, can be a promising way for selective elimination of cancer cells [97].

Higher hydroxylated resveratrol analogs (HHRA), i.e., 3,3′,4,4′–tetrahydroxy–*trans*–stilbene, 3,4,4′,5–tetrahydroxy–*trans*–stilbene and 3,3′,4,4′,5,5′–hexahydroxy–*trans*–stilbene, significantly demonstrate various biological activities when compared with resveratrol. These compounds possess both antioxidant and prooxidant properties. The antioxidant properties are expressed to enable these compounds to protect cells from oxidative damage, whereas prooxidant activity are shown by their cytotoxic or pro-apoptotic effects. These compounds exerted the cytotoxic effect on leukemia cells. Moreover, it was observed that there was increased activity of caspase 3 and 9. Cell death was accompanied by a decrease of mitochondrial potential, oxidative stress, loss of reduced glutathione level, and the diminishing of both mRNA expression and activity of MnSOD. Manganese superoxide dismutase is a key antioxidant enzyme that protects the mitochondrial matrix against the superoxide radical produced by respiratory chain activity. Also, MnSOD protects cancer cells from apoptosis induced by various pro-apoptotic agents like TNF and anticancer drugs. HHRA possessing *ortho*-hydroxy groups are stronger cytotoxic agents than compounds without such a structure. It was found that the increased cytotoxicity of *ortho*-hydroxystilbenes is associated with the presence of *ortho*-semiquinones formed during metabolism or oxidation. It was suggested that the cytotoxic activity of the tested compounds may be connected with the formation of short-living prooxidative metabolites, which cause the induction of oxidative stress in cancer cells [98, 99].

Piceatannol (3,3′,4′,5–*trans*–tetrahydroxystilbene is a naturally occurring analog of resveratrol. Piceatannol has been found in various plants, including grapes, passion fruit, and white tea. This compound exhibits antioxidative activity, and anticancer properties expressed by its ability to suppress proliferation among other breast cancers. The inhibition of cancer cells’ growth and apoptotic activities of piceatannol are mediated through cell cycle arrest; up-regulation of Bid, Bax, Bik, Bok, Fas, p21; down-regulation of Bcl-xL, BCL-2, cIAP, activation of caspases -3, -7, -8, -9, loss of mitochondrial potential, and release of cytochrome c. Piceatannol suppresses the activation of some transcription factors, including NF-кB, which plays a main role as a transcriptional regulator in response to oxidative stress caused by free radicals, cytokines, or microbial antigens. Moreover, piceatannol inhibits Janus kinase (JAK-1), which is a key factor of the STAT pathway that is crucial in controlling cellular activities in response to extracellular cytokines and is a COX-2-inducible enzyme involved in carcinogenesis. Piceatannol exerts apoptotic action but may also show anti-apoptotic and pro-proliferative activity. Piceatannol binds estrogen receptors and stimulates growth of estrogen-dependent cancer cells. The pharmacological properties of piceatannol, particularly its antitumor, antioxidant, and anti-inflammatory actions, indicate that this compound might be a potentially useful nutritional and therapeutic agent [100].

The protective anticancer effect of biologically active compounds may be realized by direct impact on the activity of the metabolized enzymes and E_2_ biotransformation.

### Chrysoeriol

Chrysoeriol (luteorin–3′–methoxyether) is a natural methoxyflavonoid that selectively inhibits CYP1B1 activity and prevents the formation of carcinogenic 4-OHE_2_ from E_2_. Chrysoeriol inhibited E_2_ hydroxylation catalyzed by CYP1B1, but not by CYP1A1. Methylation of 4-OHE_2_, which is thought to be a detoxification process, was not affected by the chrysoeriol. In MCF-7 human breast cancer cells, chrysoeriol did not affect the CYP1A1 and CYP1B1 gene expression, but significantly inhibited the production of 4-MeOE_2_ without any effects on the formation of 2-MeOE_2_ [101].

A similar effect was observed in MCF-10F cells treated with resveratrol. Resveratrol has anticancer activity because it is the aryl hydrocarbon receptor (AhR) antagonist that decreases CYP1B1 expression and induces NQO1 expression in a dose- and time-dependent manner. While CYP1B1 favors qauinone formation by catalyzing estrogen 4-hydroxylation, NQO1 catalyzes the protective reduction of quinones to catechols. Resveratrol decreased estrogen metabolism and blocked formation of DNA adducts in MCF-10F cells. Thus, resveratrol can prevent breast cancer initiation by blocking multiple stages in the estrogen genotoxicity pathway [102].

The antioxidants, N-acetylcysteine, and reduced form lipoic acid showed a significant inhibition of the formation of the depurinating estrogen-DNA adducts, N3Ade and N7Gua, by E_2_–3,4–Q. In the reaction of lactoperoxidase-activated 4-OHE_2_ with DNA, resveratrol achieved the highest level of inhibition, N-acetylcysteine and reduced lipoic acid caused moderate inhibition, and melatonin had the least inhibition. These data indicate that all four antioxidants can inhibit the formation of the depurinating estrogen-DNA adducts and to prevent the indication of human breast cancer [103].

## Conclusions

This review presents data on important findings concerning the risk factors, estrogen metabolism, and pathomechanism of breast cancer, as well as its chemoprevention. The initiation and development of breast cancer is caused by numerous risk factors.

The parent estrogen, mainly E_2_ and its metabolites, are responsible for the formation of neoplastic alterations in breast epithelial cells. Reactive oxygen species and free radicals play a significant role in the induction oncogenesis. The mechanisms of estrogen carcinogenesis include participation of E_2_ as an estrogen receptors’ agonist, the genotoxic effects of the estrogen metabolites, and epigenetic processes. The carcinogenic process occurs with the participation of numerous signaling pathways. Phytoestrogens, classical antioxidants, and some synthetic compounds, are considered to have a number of beneficial activities, including anticancer, anti-oxidative, anti-inflammatory and estrogenic activity. These chemicals suppress proliferation of a wide variety of tumor cells, including breast cancer cells. The growth-inhibitory and apoptotic effects of these compounds are mediated through cell-cycle arrest, upregulation and/or down-regulation of numerous biomolecules, activation of caspases, and loss of mitochondrial potential. These compounds have been shown to suppress the activation of some transcription factors, including NF-кB, which plays a central role as a transcriptional regulator in response to cellular stress caused by ROS and free radicals. The protective anticancer effect may be realized by direct impact of biologically active compounds to the metabolized enzymes’ activity and E_2_ biotransformation. Higher hydroxylated polyphenols possess both antioxidant and prooxidant properties. *Ortho*-hydroxystilbenes are a promising agent for selective elimination of cancer cells. The biological activity, especially phytoestrogens, suggests that these compounds might be potentially useful nutritional and chemopreventive agents.
